# Pharmacological treatment of major depressive disorder according to severity in psychiatric inpatients: results from the AMSP pharmacovigilance program from 2001–2017

**DOI:** 10.1007/s00702-022-02504-6

**Published:** 2022-05-07

**Authors:** Johanna Seifert, Hannah B. Maier, Fabienne Führmann, Stefan Bleich, Susanne Stübner, Marcel Sieberer, Xueqiong Bernegger, Waldemar Greil, Cornelius Schüle, Sermin Toto, Renate Grohmann, Matthias A. Reinhard

**Affiliations:** 1 Department of Psychiatry, Social Psychiatry and Psychotherapy, Hannover Medical School, Carl-Neuberg-Straße 1, 30625 Hannover, Germany; 2 Department of Psychiatry and Psychotherapy, KRH Psychiatrie GmbH, Wunstorf, Germany; 3 Department of Psychiatry and Psychotherapy, LMU University Hospital Munich, Munich, Germany; 4 Department of Forensic Psychiatry, Bezirksklinikum Ansbach, Ansbach, Germany; 5 Department of Psychiatry, Psychotherapy and Psychosomatics, St. Marien-Hospital Hamm gGmbH, Hamm, Germany; 6 Department of Psychiatry and Psychotherapy, University Witten/Herdecke, Witten, Germany; 7 Psychiatric Private Hospital, Sanatorium Kilchberg, Kilchberg, Switzerland

**Keywords:** Major depressive disorder, Pharmacotherapy, Antidepressant drugs, Antipsychotic drugs, Psychiatric inpatients, Pharmacovigilance

## Abstract

The International Classification of Diseases (10^th^ Version) categorizes major depressive disorder (MDD) according to severity. Guidelines provide recommendations for the treatment of MDD according to severity. Aim of this study was to assess real-life utilization of psychotropic drugs based on severity of MDD in psychiatric inpatients. Drug utilization data from the program “Drug Safety in Psychiatry” (German: Arzneimittelsicherheit in der Psychiatrie, AMSP) were analyzed according to the severity of MDD. From 2001 to 2017, 43,868 psychiatric inpatients with MDD were treated in participating hospitals. Most patients were treated with ≥ 1 antidepressant drug (ADD; 85.8% of patients with moderate MDD, 89.8% of patients with severe MDD, and 87.9% of patients with psychotic MDD). More severely depressed patients were more often treated with selective serotonin–norepinephrine reuptake inhibitors and mirtazapine and less often with selective serotonin reuptake inhibitors (*p* < 0.001 each). Use of antipsychotic drugs (APDs), especially second-generation APDs, increased significantly with severity (37.0%, 47.9%, 84.1%; *p* < 0.001 each). APD + ADD was the most used combination (32.8%, 43.6%, 74.4%), followed by two ADDs (26.3%, 29.3%, 24.9%). Use of lithium was minimal (3.3%, 6.1% ,7.1%). The number of psychotropic drugs increased with severity of MDD—patients with psychotic MDD had the highest utilization of psychotropic drugs (93.4%, 96.5%, 98.7%; *p* < 0.001). ADD monotherapy was observed to a lesser extent, even in patients with non-severe MDD (23.2%, 17.1%, 4.4%). Findings reveal substantial discrepancies between guideline recommendations and real-life drug utilization, indicating that guidelines may insufficiently consider clinical needs within the psychiatric inpatient setting.

## Introduction

Worldwide, the incidence of depression has increased by nearly 50% within the past 3 decades (Liu et al. 2019) which is one of the most common serious medical and psychiatric disorders (Rush 2007). The lifetime prevalence of MDD is estimated at 14.6% in high-income countries and 11.1% in low- to middle-income countries, whereas the 12-month prevalence is reported as 5.5% in high-income countries and 5.9% in low- to middle-income countries (Bromet et al. 2011). The severity of symptoms is associated with increased morbidity, all-cause mortality, functional impairment, and disability (Lépine and Briley 2011), resulting in high direct and indirect costs of depression (König et al. 2019).

Clinical presentation of major depressive disorder (MDD) is variable and affects a heterogeneous group of patients (Henkel et al. 2011). Different “subtypes” of depression have been determined based on different symptom presentation (e.g., atypical, melancholic, psychotic, and anxious), onset characteristics (e.g., post-partum, seasonal, and early vs. late-onset), course of illness (e.g., single or recurrent episode, chronic), and severity (e.g., mild, moderate, and severe). The latter classification is widely used in clinical settings (Rush 2007) and in the conceptualization of diagnosis according to the International Classification of Disease, 10th Version (ICD-10) (WHO 1992). The most debilitating subtype is severe MDD with psychotic symptoms (in the following referred to as *psychotic MDD*), which is associated with a high risk of recurrence and mortality (Jääskeläinen et al. 2018).

Numerous studies have suggested that baseline severity of MDD corresponds with the outcome of antidepressant treatment (Henkel et al. 2011; Khan et al. 2002, 2005; Kirsch et al. 2008). While patients with more severe symptoms are more likely to respond to antidepressant treatment, those who are less severely ill may respond equally well to placebo (Khan et al. 2002). Moreover, it has been proposed that patients with non-severe depression show no benefit when treated with antidepressant drugs (ADDs) (Kirsch et al. 2008)—a matter of fierce debate (Stewart et al. 2012).

Official treatment guidelines such as those by the American Psychiatric Association (APA) (Gelenberg et al. 2010), the National Institute for Health and Clinical Excellence (NICE) (NICE 2009), the World Federation of Societies of Biological Psychiatry (WFSBP) (Bauer et al. 2013), and the German S3 guideline from 2015 (DGPPN et al. 2015) use the degree of severity of MDD to guide treatment. Within these guidelines, recommendations based on the severity of depression are largely consistent for moderate and severe MDD with greater variance in recommendations for mild depression (Davidson 2010). First-line recommendations for moderate MDD include the use of ADDs, psychotherapy, or a combination of both, whereas severe MDD with and without psychotic symptoms may require a more complex regimen. The German Association for Psychiatry, Psychotherapy, and Psychosomatics (German: Deutsche Gesellschaft für Psychiatrie, Psychotherapie und Nervenheilkunde, DGPPN) treatment guidelines from 2015 specifically recommend either psychotherapy or pharmacotherapy for moderate MDD and a combination of both for severe MDD with or without psychotic symptoms. According to these guideline recommendations, treatment efficacy should be monitored once a week, and if symptoms have not sufficiently improved after 3–4 weeks, treatment should be reconsidered or augmented (DGPPN et al. 2015).

The use of ADDs in the treatment of MDD is recommended under consideration of the patient’s individual characteristics and treatment preferences in both moderate and severe MDD (NICE 2009; Bauer et al. 2013; Gelenberg et al. 2010; DGPPN et al. 2015). Selective serotonin reuptake inhibitors (SSRIs) are most widely recommended as first-line therapy due to their higher tolerability when compared to other ADDs such as tricyclic antidepressants (TCAs) (Bauer et al. 2013). About 30% of patients with MDD fail to respond to the first treatment trial (Rush et al. 2006) regardless of the ADD chosen (Bauer et al. 2013). After the first unsuccessful treatment trial, there are several options for further action including (a) increasing the dosage up to the maximum dose possible of the initial ADD, (b) switching to another ADD from a different substance class or within the same substance class (this approach is only recommended if at least some response to the initial ADD was observed), (c) combination of two ADDs (e.g., SSRI/selective serotonin-norepinephrine reuptake inhibitor [SSNRI] plus noradrenergic and specific serotonergic antidepressant [NaSSA]), (d) augmentation with non-ADDs such as lithium, antipsychotic drugs (APDs) or thyroid hormones to amplify the ADD’s efficacy, (e) if not already established: psychotherapy, or (f) combining the ADD with non-pharmacological treatment options such as electroconvulsive therapy (ECT) (Bauer et al. 2013). The use of benzodiazepines receives only limited value as short-term adjunctive therapy under certain circumstances such as catatonic depression or acute suicidality (Davidson 2010).

This study aims to assess the use of individual and combinations of psychotropic drugs used in the treatment of patients suffering from MDD according to the severity of MDD in a real-life clinical inpatient setting from 2001 to 2017.

## Methods

### Data source

Data on psychotropic drug use in the treatment of patients with MDD were collected by the European program “Drug Safety in Psychiatry” (German: “Arzneimittelsicherheit in der Psychiatrie”, AMSP). AMSP was founded in 1993 and has since gathered data on psychotropic drug use and severe adverse drug reactions (ADRs) from psychiatric hospitals within a real-life setting. The number of participating hospitals has increased from nine in 1994 to 52 psychiatric institutions in Germany, Austria, and Switzerland in 2017.

All hospitals participating in the AMSP project gather drug use data, including exact dose of all psychotropic drugs for all inpatients currently in treatment on two reference days per year. Further information on age, sex, as well as psychiatric and somatic illnesses of patients is documented. Due to the inpatient setting, AMSP assesses actual utilization rates of psychotropic drugs versus merely prescription rates. A more detailed description of AMSP’s methods can be found elsewhere (Grohmann et al. 2004, 2014; Engel et al. 2004).

### Study population and design

All patients treated between 2001 and 2017 aged 18–100 years with a primary psychiatric diagnosis of MDD were included in this analysis. A systematic documentation of psychiatric and somatic comorbidities has only been performed since 2008 and therefore comorbidities not considered during data analysis. MDD was identified using the International Classification of Disease in its 10^th^ Version (ICD–10) categorized as mild (F32.0, F32.00, F32.01, F33.0, F33.00, F33.01), moderate (F32.1, F32.10, F32.11, F33.1, F33.10, F33.11), or severe without (F32.2, F33.2) or with psychotic symptoms (F32.3, F33.3) (WHO 1992). Patients suffering from mild depression were excluded from further analysis due to the very low rate of patients (< 2% of all patients) and, therefore, the limited validity of potential conclusions. To improve readability of the manuscript, severe MDD without psychotic symptoms will be referred to as “severe MDD” and severe MDD with psychotic symptoms as “psychotic MDD”.

### Classification of psychotropic drugs and psychotropic drug use

Table 1 shows the classification of psychotropic drug groups (i.e., ADDs, APDs, hypnotic drugs [HYPDs], tranquilizing drugs [TRDs], and antiepileptic drugs [AEDs]) including individual drugs relevant to this study. The term “monotherapy” refers to the use of one psychotropic drug without concomitant use of any other psychotropic drug and “polypsychopharmacotherapy” is defined as the utilization of more than two psychotropic drugs of different drug groups (Masnoon et al. 2017). Due to the unavailability of clinical data, it is not possible to assess whether additional psychotropic drugs were prescribed as an augmentation strategy due to insufficient efficacy of a previously used ADD (Bauer et al. 2013) or to treat a specific symptom such as restlessness. In the following, the use of more than one psychotropic drug will be referred to as a “combination of psychotropic drugs”, which includes the utilization of additional psychotropic drugs as an augmentation strategy.

**Table 1:** Classification of psychotropic drugs relevant to this study

Psychotropic drug group		Subgroup	Individual drugs
ADD		*SSRI*	escitalopram, citalopram, sertraline*
		*SSNRI*	venlafaxine, duloxetine*
		*TCA*	trimipramine, amitriptyline, doxepin*
		*NaSSA*	mirtazapine*
		*MAO-I*	tranylcypromine, moclobemide**
		*other ADD*	trazodone, bupropion, agomelatine*
APD	FGA	lp FGA	pipamperone, promethazine, prothipendyl, melperone*
		hp FGA	haloperidol, perazine, flupentixol**
	SGA		quetiapine, olanzapine, risperidone*
	TGA		aripiprazole*
HYPD		Z-drug	zopiclone, zolpidem*
TRD		benzodiazepine	lorazepam, diazepam, oxazepam*
AED			valproic acid, lamotrigine, and pregabalin*

*ADD* antidepressant drug, *SSRI* selective serotonin reuptake inhibitor, *SSNRI* selective serotonin–norepinephrine reuptake inhibitor, *TCA* tricyclic antidepressant, *NaSSA* noradrenergic and specific serotonergic antidepressant, *MAO*-*I* monoamine oxidase inhibitor, *APD* antipsychotic drug, *FGA* first-generation antipsychotic drug, *lp* low potency, *hp* high potency, *SGA* second-generation antipsychotic drug, *TGA* third-generation antipsychotic drug, *HYPD* hypnotic drug, *TRD* tranquilizing drug, *AED* antiepileptic drug

*only drugs used in the treatment of ≥ 2.5% of patients are listed

**because of very low overall utilization in these drug groups, drugs used in the treatment of ≥ 0.5% of patients are listed

### Statistical analysis

Data were analyzed with IBM SPSS^©^ Version 25.0 and Excel^©^ 2019. Descriptive data are presented separately for the three MDD severity groups as mean and standard deviation (SD) or as percentages. Percentages refer to the total number of patients suffering from a specific severity of MDD. As aripiprazole was not available prior to 2007, only data from 2007 to 2017 are shown.

In case of explicit drug combinations, percentages refer to the number of patients treated with the first-named drug/drug group/subgroup. Chi-square tests with three groups were performed to detect significant overall group differences in drug use. Because of the large sample size, it is a statistic certainty that even small or marginal differences without clinical relevance appear "significant". Therefore, effect sizes are reported as Cramer’s V (0.07 indicates a small, 0.21 a medium, and 0.35 a large effect in case of df = 2) or as Phi (ϕ; 0.1 indicates a small, 0.3 a medium, and 0.5 a large effect). Furthermore, one-way ANOVAs with Bonferroni corrected post hoc tests were used to compare the number of psychotropic drugs between groups. Effect sizes are reported as eta squared (η^2^; 0.01 indicates a small, 0.06 a medium, and 0.14 a large effect).

## Results

### Characteristics of the study sample

A total of 43,868 inpatients (62.8% female, mean age: 50.4 years) with a diagnosis of moderate, severe, or psychotic MDD were identified in the AMSP database which comprises a total of 147,481 patients. Among patients with MDD, 28.1% suffered from moderate MDD (N = 12,316, 63.1% female, mean age: 47.9 years), 59.2% from severe MDD (N = 25,962, 62.9% female, mean age: 50.7 years), and 12.7% from psychotic MDD (N = 5,590, 61.9% female, mean age: 54.9 years; Table 2). Table 2 also shows the percentage of patients according to severity of MDD within each time frame.

**Table 2:** Sample description and proportion of severity degrees of major depressive disorder (MDD) for different time periods in the study sample (in % of all MDD patients; in brackets: absolute number of patients)

	Moderate MDD	Severe MDD	Psychotic MDD
Total *N*	28.1% (12,316)	59.2% (25,962)	12.7% (5,590)
2001–2005	31.8% (2,693)	51.9% (4,388)	16.3% (1,377)
2006–2009	27.5% (2,490)	57.9% (5,246)	14.6% (1,322)
2010–2013	26.5% (3,394)	62.2% (7,956)	11.2% (1,437)
2014–2017	27.4% (3,739)	61.7% (8,372)	10.7% (1,454)
Female	63.1% (7,771)	62.9% (16,330)	61.9% (3,460)
Mean age	47.9 years	50.7 years	54.9 years

### Utilization rates of psychotropic drugs according to severity of MDD

#### Utilization rates of ADDs, APDs, AEDs, and lithium

**Fig. 1:**
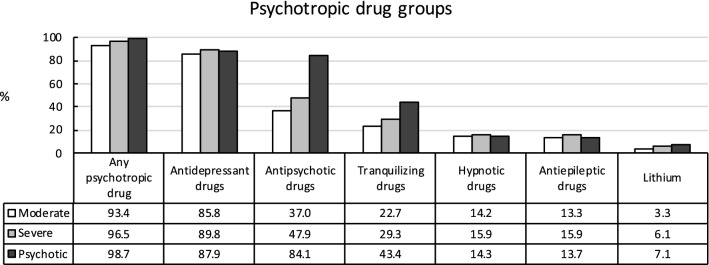
Utilization of main groups of psychotropic drugs depending on severity of major depressive disorder. Any psychotropic drug: overall: X^2^(2,43,868) = 331.8, *p*< 0.001, *V*= 0.06; moderate vs. severe: X^2^(1,38,278) = 186.9, *p*< 0.001, ϕ = 0.07; moderate vs. psychotic: X^2^(1,17,906) = 229.8, *p*< 0.001, ϕ = 0.11; severe vs. psychotic: X^2^(1,31,552) = 73.9, *p*< 0.001, ϕ = 0.05. Antidepressant drugs: overall: X^2^(2,43,868) = 132.5, *p*< 0.001, *V*= 0.04; moderate vs. severe: X^2^(1,38,278) = 131.5, *p*< 0.001, ϕ = 0.06; moderate vs. psychotic: X^2^(1,17,906) = 145, *p*< 0.001, ϕ = 0.03; severe vs. psychotic: X^2^(1,31,552) = 167.6, *p*< 0.001, ϕ = 0.02. Antipsychotic drugs: overall: X^2^(2,43,868) = 3473.6, *p*< 0.001, *V*= 0.20; moderate vs. severe: X^2^(1,38,278) = 402.0, *p*< 0.001, ϕ = 0.10; moderate vs. psychotic: X^2^(1,17,906) = 3415.8, *p*< 0.001, ϕ = 0.44; severe vs. psychotic: X^2^(1,31,552) = 2429.1, *p*< 0.001, ϕ = 0.28. Tranquilizing drugs: overall: X^2^(2,43,868) = 796.3, *p*< 0.001, *V*= 0.10; moderate vs. severe: X^2^(1,38,278) = 183.9, *p*< 0.001, ϕ = 0.07; moderate vs. psychotic: X^2^(1,17,906) = 797.5, *p*< 0.001, ϕ = 0.21; severe vs. psychotic: X^2^(1,31,552) = 421.7, *p*< 0.001, ϕ = 0.12. Hypnotic drugs: overall: X^2^(2,43,868) = 22.9, *p*< 0.001, *V*= 0.02; moderate vs. severe: X^2^(1,38,278) = 18.6, *p*< 0.001, ϕ = 0.02; moderate vs. psychotic: X^2^(1,17,906) = 0.1, *p*= 0.86, ϕ < 0.01; severe vs. psychotic: X^2^(1,31,552) = 8.9, *p*< 0.05, ϕ = 0.02. Antiepileptic drugs: overall: X^2^(2,43,868) = 51.7, *p*< 0.001, *V*= 0.02; moderate vs. severe: X^2^(1,38,278) = 44.1, *p*< 0.001, ϕ = 0.03; moderate vs. psychotic: X^2^(1,17,906) = 0.5, *p*= 0.42, ϕ = 0.01; severe vs. psychotic: X^2^(1,31,552) = 17.0, *p*< 0.001, ϕ = 0.02. Lithium**:** overall: X^2^(2,43,868) = 161.5, *p*< 0.001, *V*= 0.04; moderate vs. severe: X^2^(1,38,278) = 132.9, *p*< 0.001, ϕ = 0.06; moderate vs. psychotic: X^2^(1,17,906) = 129.6, *p*< 0.001, ϕ = 0.09; severe vs. psychotic: X^2^(1,31,552) = 7.8, *p*< 0.05, ϕ = 0.02

Utilization rates of psychotropic drugs increased with MDD severity (moderate MDD: 93.4%, severe MDD: 96.5%, psychotic MDD: 98.7%; see Fig. 1). While a majority of patients with MDD received ADDs, ADD utilization rates differed significantly between groups (85.8%, 89.8%, and 87.9%). Utilization rates of APDs (37.0%, 47.9%, and 84.1%) and TRDs (22.7%, 29.3%, and 43.4%) significantly increased with severity of MDD. Furthermore, patients with severe MDD were more likely to be treated with HYPDs (14.2%, 15.9%, and 14.3%) than patients with moderate MDD or psychotic MDD, whereas moderate and psychotic MDD did not differ. AEDs were more often used in severe MDD than moderate MDD and psychotic MDD (13.3%, 15.9%, and 13.7%). Again, moderate and psychotic MDD did not differ regarding AED rates. Finally, the use of lithium significantly increased with severity of MDD (3.3%, 6.1%, and 7.1%), but was low in all three groups.

#### Utilization rates of ADD and APD subgroups

**Fig. 2:**
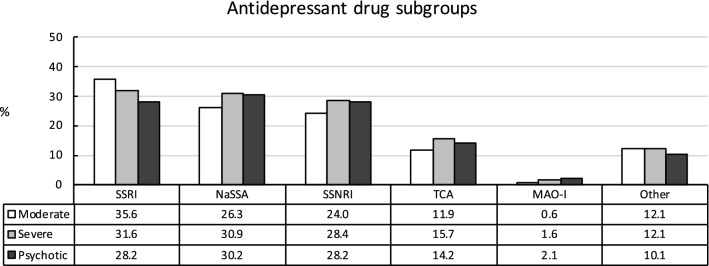
Utilization of subgroups of antidepressant drugs depending on severity of major depressive disorder. **SSRI**: selective serotonin reuptake inhibitor; overall: X^2^(2,43,868) = 110.1, *p*< 0.001, *V*= 0.04; moderate vs. severe: X^2^(1,38,278) = 60.6, *p*< 0.001, ϕ = 0.04; moderate vs. psychotic: X^2^(1,17,906) = 94.8, *p*< 0.001, ϕ = 0.07; severe vs. psychotic: X^2^(1,31,552) = 24.9, *p*< 0.001, ϕ = 0.03. NaSSA: noradrenergic and specific serotonergic antidepressant: overall: X^2^(2,43,868) = 86.4, *p*< 0.001, *V*= 0.03; moderate vs. severe: X^2^(1,38,278) = 85.1, *p*< 0.001, ϕ = 0.05; moderate vs. psychotic: X^2^(1,17,906) = 29.7, *p*< 0.001, ϕ = 0.04; severe vs. psychotic: X^2^(1,31,552) = 1.1, *p*= 0.30, ϕ = 0.01. SSNRI: selective serotonin-norepinephrine reuptake inhibitor: overall: X^2^(2,43,868) = 85.4, *p*< 0.001, *V*= 0.03; moderate vs. severe: X^2^(1,38,278) = 82.1, *p*< 0.001, ϕ = 0.05; moderate vs. psychotic: X^2^(1,17,906) = 35.9, *p*< 0.001, ϕ = 0.04; severe vs. psychotic: X^2^(1,31,552) = 0.1, *p*= 0.76, ϕ < 0.01. TCA: tricyclic antidepressant: overall: X^2^(2,43,868) = 97.9, *p*< 0.001, *V*= 0.03; moderate vs. severe: X^2^(1,38,278) = 97.4, *p*< 0.001, ϕ = 0.05; moderate vs. psychotic: X^2^(1,17,906) = 18.4, *p*< 0.001, ϕ = 0.03; severe vs. psychotic: X^2^(1,31,552) = 7.9, *p*< 0.05, ϕ = 0.02. MAO-I: monoamine oxidase inhibitor: overall: X^2^(2,43,868) = 85.4, *p*< 0.001, *V*= 0.03; moderate vs. severe: X^2^(1,38,278) = 66.2, *p*< 0.001, ϕ = 0.04; moderate vs. psychotic: X^2^(1,17,906) = 81.9, *p*< 0.001, ϕ = 0.07; severe vs. psychotic: X^2^(1,31,552) = 6.9, *p*< 0.05, ϕ = 0.01

The use of SSRIs significantly decreased with depression severity (35.6%, 31.6%, and 28.2%; see Fig. 2). In contrast, patients suffering from severe and psychotic MDD were more often treated with NaSSAs (26.3%, 30.9%, and 30.2%) and SSNRIs (24.0%, 28.4%, and 28.2%) than patients with moderate MDD. Treatment with SSNRIs and NaSSAs did not differ significantly between patients with severe or psychotic MDD. Utilization of TCAs was more common in severe MDD than moderate or psychotic MDD (11.9%, 15.7%, and 14.2%) and less common in moderate than psychotic MDD. The use of MAO-Is significantly increased with MDD severity (0.6%, 1.6%, and 2.1%), but was very low in all three groups.

**Fig. 3:**
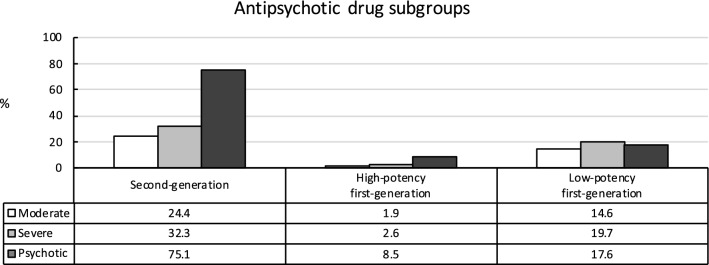
Utilization of subgroups of antipsychotic drugs (APD) depending on severity of major depressive disorder. Second-generation APD: X^2^(2,43,868) = 4605.1, *p*< 0.001, *V*= 0.23; moderate vs. severe: X^2^(1,38,278) = 249.4, *p*< 0.001, ϕ = 0.08; moderate vs. psychotic: X^2^(1,17,906) = 4110.3, *p*< 0.001, ϕ = 0.48; severe vs. psychotic: X^2^(1,31,552) = 3514.2, *p*< 0.001, ϕ = 0.33. High-potency (hp) first-generation APDs: overall: X^2^(2,43,868) = 612.3, *p*< 0.001, *V*= 0.08; moderate vs. severe: X^2^(1,38,278) = 17.7, *p*< 0.001, ϕ = 0.02; moderate vs. psychotic: X^2^(1,17,906) = 440.3, *p*< 0.001, ϕ = 0.16; severe vs. psychotic: X^2^(1,31,552) = 455.9, *p*< 0.001, ϕ = 0.12. Low-potency (lp) first-generation APDs: overall: X^2^(2,43,868) = 147.9, *p*< 0.001, *V*= 0.04; moderate vs. severe: X^2^(1,38,278) = 146.8, *p*< 0.001, ϕ = 0.06; moderate vs. psychotic: X^2^(1,17,906) = 26.4, *p*< 0.001, ϕ = 0.04; severe vs. psychotic: X^2^(1,31,552) = 13.0, *p*< 0.001, ϕ = 0.02

The most utilized subgroup of APDs by far was second-generation APDs (SGAs). Treatment with SGAs significantly increased with the level of severity of MDD (24.4%, 32.3%, and 75.1%) and was particularly high among patients suffering from psychotic MDD (see Fig. 3). Similarly, high-potency (hp) FGAs were significantly more often used with increasing MDD severity (1.9%, 2.6%, and 8.5%), however, their use remained below 10% even among patients with psychotic MDD. Finally, patients suffering from severe MDD showed higher treatment rates with low-potency (lp) FGAs than patients with psychotic and moderate MDD (14.6%, 19.7%, 17.6%). In addition, lp FGA rates were higher in psychotic MDD than in moderate MDD.

Table 3 shows individual psychotropic drugs, median dose, and their use according to severity of MDD. The median dosages of SSRIs, SSNRIs, TCAs, and quetiapine increased with MDD severity.

**Table 3:** Utilization rate and median dosage of the most frequently (i.e., > 2.5% of patients) used antidepressant (ADD), antipsychotic (APD), hypnotic (HYPD), tranquilizing (TRD), and antiepileptic drugs (AED) according to severity of major depressive disorder (MDD)

Substance	Moderate MDD		Severe MDD		Psychotic MDD		Overall:	Moderate vs. severe:	Moderate vs. psychotic:	Severe vs. psychotic:
	Utilization rate	Median [mg]	Utilization rate	Median [mg]	Utilization rate	Median [mg]	X^2^(2,43,868)	X^2^(1,38,278)	X^2^(1,17,906)	X^2^(1,31,552)
ADD										
Mirtazapine	23.4%	30	28.0%	30	27.5%	30	92.8, *p* <0.001, *V* = 0.03	90.7, *p* <0.001, ϕ = 0.05	34.8, *p* <0.001, ϕ = 0.04	0.6, *p* <0.001, ϕ < 0.01
Venlafaxine	17.6%	150	21.6%	150	22.3%	225	93.6, *p* <0.001, *V* = 0.03	82.6, *p* <0.001, ϕ = 0.05	55.0, *p* <0.001, ϕ = 0.06	1.3, *p* =0.25, ϕ = 0.01
Escitalopram	11.4%	10	9.9%	15	9.1%	15	29.2, *p* <0.001, *V* = 0.02	20.2, *p* <0.001, ϕ = 0.02	21.3, *p* <0.001, ϕ = 0.03	3.3, *p* <0.001, ϕ = 0.01
Citalopram	9.8%	20	9.6%	20	7.3%	30	32.5, *p* <0.001, *V* = 0.02	0.4, *p* <0.001, ϕ < 0.01	29.3, *p* <0.001, ϕ = 0.04	29.1, *p* <0.001, ϕ = 0.03
Sertraline	8.8%	100	8.6%	100	8.4%	100	0.9, *p* =0.65, *V* < 0.01	0.4, *p* =0.52, ϕ < 0.01	0.8, *p* =0.38, ϕ = 0.01	0.2, *p* =0.63, ϕ < 0.01
Duloxetine	7.9%	60	8.7%	60	7.3%	90	15.4, *p* <0.001, *V* = 0.01	6.9, *p* <0.05, ϕ = 0.01	1.9, *p* =0.16, ϕ = 0.01	11.7, *p* <0.001, ϕ = 0.02
Trazodone	8.3%	150	4.8%	150	5.5%	150	186.7, *p* <0.001, *V* = 0.05	183.6, *p* <0.001, ϕ = 0.07	43.8, *p* <0.001, ϕ = 0.05	4.8, *p* <0.05, ϕ = 0.01
Trimipramine	4.9%	75	4.3%	75	3.0%	100	33.7, *p* <0.001, *V* = 0.02	7.0, *p* <0.05, ϕ = 0.01	33.7, *p* <0.001, ϕ = 0.04	19.9, *p* <0.001, ϕ = 0.03
Amitriptyline	2.7%	75	4.4%	100	4.5%	100	69.3, *p* <0.001, *V* = 0.03	65.2, *p* <0.001, ϕ = 0.04	39.5, *p* <0.001, ϕ = 0.05	0.1, *p* =0.74, ϕ < 0.01
Agomelatine	2.5%	25	3.1%	50	1.4%	25	53.3, *p* <0.001, *V* = 0.02	10.7, *p* <0.001, ϕ = 0.02	22.0, *p* <0.001, ϕ = 0.04	48.9, *p* <0.001, ϕ = 0.04
Bupropion	2.6%	300	3.0%	300	1.8%	150	26.1, *p* <0.001, *V* = 0.02	4.8, *p* <0.05, ϕ = 0.01	10.7, *p* <0.001, ϕ = 0.02	24.4, *p* <0.001, ϕ = 0.03
Doxepin	2.4%	75	2.9%	100	2.1%	100	15.8, *p* <0.001, *V* = 0.01	7.8, *p* <0.05, ϕ = 0.01	1.5, *p* =0.22, ϕ = 0.01	11.0, *p* <0.001, ϕ = 0.02
APD										
Quetiapine	14.3%	100	17.6%	150	24.7%	200	287.1, *p* <0.001, *V* = 0.06	65.9, *p* <0.001, ϕ = 0.04	287.4, *p* <0.001, ϕ = 0.13	151.5, *p* <0.001, ϕ = 0.07
Olanzapine	4.7%	7.5	7.3%	7.5	22.9%	10	1756.1, *p* <0.001, *V* = 0.14	93.4, *p* <0.001, ϕ = 0.05	1368.9, *p* <0.001, ϕ = 0.28	1236.7, *p* <0.001, ϕ = 0.20
Risperidone	3.1%	2	4.9%	2	20.7%	2	2227.0, *p* <0.001, *V* = 0.16	65.5, *p* <0.001, ϕ = 0.04	1516.1, *p* <0.001, ϕ = 0.29	1615.8, *p* <0.001, ϕ = 0.23
Pipamperone	3.7%	40	5.9%	40	4.7%	40	85.5, *p* <0.001, *V* = 0.03	82.1, *p* <0.001, ϕ = 0.05	10.0, *p* <0.001, ϕ = 0.02	12.3, *p* <0.001, ϕ = 0.02
Prothipendyl	3.0%	80	4.2%	80	4.0%	80	33.1, *p* <0.001, *V* = 0.02	32.8, *p* <0.001, ϕ = 0.03	12.0, *p* <0.001, ϕ = 0.03	0.5, *p* =0.50, ϕ < 0.01
Melperone	1.9%	50	3.0%	50	3.1%	50	42.2, *p* <0.001, *V* = 0.02	39.2, *p* <0.001, ϕ = 0.03	24.9, *p* <0.001, ϕ = 0.04	0.2, *p* =0.69, ϕ < 0.10
Aripiprazole	1.4%	10	2.5%	10	5.5%	10	259.2, *p* <0.001, *V* = 0.05	48.1, *p* <0.001, ϕ = 0.04	247.8, *p* <0.001, ϕ = 0.12	140.8, *p* <0.001, ϕ = 0.07
HYPD										
Zopiclone	4.9%	7.5	8.3%	7.5	5.8%	7.5	162.6, *p* <0.001, *V* = 0.04	144.4, *p* <0.001, ϕ = 0.06	6.3, *p* <0.05, ϕ = 0.02	39.7, *p* <0.001, ϕ = 0.04
Zolpidem	5.1%	10	4.5%	10	5.1%	10	8.4, *p* <0.05, *V* = 0.01	6.7, *p* <0.05, ϕ = 0.01	0.0, *p* =0.99., ϕ = 0.00	3.8, *p* = 0.05, ϕ = 0.01
TRD										
Lorazepam	12.8%	1.75	19.8%	1.5	32.7%	1.5	975.0, *p* <0.001, *V* = 0.11	282.9, *p* <0.001, ϕ = 0.09	988.9, *p* <0.001, ϕ = 0.23	444.8, *p* <0.001, ϕ = 0.12
Diazepam	2.1%	9.5	3.6%	7.0	4.7%	10	96.6, *p* <0.001, *V* = 0.03	62.2, *p* <0.001, ϕ = 0.04	91.9, *p* <0.001, ϕ = 0.07	15.2, *p* <0.001, ϕ = 0.02
Promethazine	3.1%	50	4.4%	50	3.1%	50	48.1, *p* <0.001, *V* = 0.02	36.9, *p* <0.001, ϕ = 0.03	0.0, *p* =0.99, ϕ = 0.00	19.5, *p* <0.001, ϕ = 0.02
Oxazepam	2.4%	30	2.9%	22.5	3.7%	25	23.7, *p* <0.001, *V* = 0.02	7.8, *p* <0.05, ϕ = 0.01	23.8, *p* <0.001, ϕ = 0.04	10.0, *p* <0.05, ϕ = 0.02
AED										
Pregabalin	4.0%	150	5.8%	175	4.0%	150	71.4, *p* <0.001, *V* = 0.03	54.7, *p* <0.001, ϕ = 0.04	0.0, *p* =0.99, ϕ = 0.00	28.8, *p* <0.001, ϕ = 0.03
Lamotrigine	2.4%	100	3.3%	100	3.2%	125	23.6, *p* <0.001, *V* = 0.02	23.2, *p* <0.001, ϕ = 0.02	9.5, *p* <0.05, ϕ = 0.02	0.1, *p* =0.70, ϕ < 0.01
Valproat	2.6%	1000	2.7%	1000	3.0%	1000	2.4, *p* =0.31, *V* = 0.01	0.3, *p* =0.57, ϕ < 0.01	2.3, *p* =0.13, ϕ = 0.01	1.5, *p* =0.21, ϕ = 0.01

### Monotherapy and number of psychotropic drugs used according to severity of MDD

ADD monotherapy decreased with severity of MDD (23.2%, 17.1%, and 4.4%; see Table 4). Among ADD subgroups SSRIs were the ADD subgroup most commonly used in ADD monotherapy (9.3%, 6.1%, and 1.5%) followed by NaSSAs (5.3%, 4.3%, and 1.2%) and SSNRIs (5.0%, 3.7%, and 0.8%). Monotherapy with TCAs was rare (1.4%, 1.7%, and 0.1%). The rate of patients with MDD treated with APD monotherapy was highest in psychotic MDD (1.9%, 1.8%, and 3.1%).

**Table 4:** Percentage of monotherapy with antidepressant (ADD) or antipsychotic drugs (APD) defined as one drug without concomitant use of any other psychotropic drug and mean number of psychotropic drugs, ADD, and APD per patient according to severity of major depressive disorder (MDD)

		Moderate MDD	Severe MDD	Psychotic MDD	Overall: X^2^(2,43,868)	Moderate vs. severe: X^2^(1,38,278)	Moderate vs. psychotic: X^2^(1,17,906)	Severe vs. psychotic: X^2^(1,31,552)
ADD monotherapy	Any	23.2%	17.1%	4.4%	954.9, *p* < 0.001, *V* = 0.10	201.5, *p* < 0.001, ϕ = 0.07	948.5, *p* < 0.001, ϕ = 0.23	586.7, *p* < 0.001, ϕ = 0.14
	SSRI	9.3%	6.1%	1.5%	400.1, *p* < 0.001, *V* = 0.07	129.2, *p* < 0.001, ϕ = 0.06	36,594, *p* < 0.001, ϕ = 0.14	194.4, *p* < 0.001, ϕ = 0.08
	NaSSA	5.3%	4.3%	1.2%	163.2, *p* < 0.001, *V* = 0.04	18.9, *p* < 0.001, ϕ = 0.02	167.5, *p* < 0.001, ϕ = 0.10	122.4, *p* < 0.001, ϕ = 0.06
	SSNRI	5.0%	3.7%	0.8%	190.6, *p* < 0.001, *V* = 0.05	35.8, *p* < 0.001, ϕ = 0.03	190.9, *p* < 0.001, ϕ = 0.10	125.4, *p* < 0.001, ϕ = 0.06
	TCA	1.4%	1.7%	0.1%	84.6, *p* < 0.001, *V* = 0.03	4.8, *p* < 0.05, ϕ = 0.01	66.0, *p* < 0.001, ϕ = 0.06	84.3, *p* < 0.001, ϕ = 0.05
APD monotherapy		1.9%	1.8%	3.1%	40.6, *p* < 0.001, *V* = 0.02	0.5, *p* = 0.50, ϕ < 0.01	24.9, *p* < 0.001, ϕ = 0.04	39.1, *p* < 0.001, ϕ = 0.04
Number of psychotropic drugs per patient	> 2	35.5%	46.3%	66.6%				
	2	31.0%	30.2%	24.3%				
	1	26.9%	20.0%	7.8%				
	0	6.6%	3.5%	1.3%				
Mean number of psychotropic drugs		2.2 ± 1.3	2.5 ± 1.4	3.1 ± 1.3	949.3, *p* < 0.001, η^2^ = 0.041	*p* < 0.001, η^2^ = 0.013	*p* < 0.001, η^2^ = 0.041	*p* < 0.001, η^2^ = 0.019
Mean number of ADDs		1.1 ± 0.7	1.2 ± 0.6	1.1 ± 0.6	66.5, *p* < 0.001 *V* < 0.001, η^2^ = 0.003	*p* < 0.001, η^2^ = 0.001	*p* = 0.42, η^2^ < 0.001	*p* < 0.001, η^2^ = 0.001
Mean number of APDs		0.4 ± 0.6	0.6 ± 0.7	1.1 ± 0.7	2003.2, *p* < 0.001, η^2^ = 0.084	*p* < 0.001, η^2^ = 0.010	*p* < 0.001, η^2^ = 0.084	*p* < 0.001, η^2^ = 0.060

The level of severity of MDD significantly affected the number of psychotropic drugs used and was lower in moderate compared to severe and psychotic MDD and higher in psychotic than in severe MDD (see Table 4). Similarly, severity of MDD was associated with the number of APDs which was highest among patients suffering from psychotic compared to moderate and severe MDD as well as higher in severe than moderate MDD. The mean number of ADDs significantly differed according to severity and was higher in severe MDD compared to moderate and psychotic MDD and did not significantly differ among moderate and psychotic MDD.

### Combinations of psychotropic drugs according to severity of MDD

Table 5 shows combinations of different psychotropic drugs, drug groups, and subgroups according to severity. The most common combination of psychotropic drugs used in patients with MDD regardless of severity was ADD + APD (32.8%, 43.6%, and 74.4%). Depending on severity of MDD, the use of combinations of individual subgroups of psychotropic drugs showed differences. SSRI were most often combined with SGAs (23.9%) among patients with moderate MDD, whereas SSNRI + SGA were particularly often utilized in patients with severe (40.0%) and psychotic MDD (79.2%). Among patients with moderate and severe MDD, quetiapine was the APD most commonly combined with an ADD (12.7% and 15.9%). The spectrum of APDs used alongside an ADD in the treatment of psychotic MDD was more variable. Olanzapine (20.5%) and risperidone (18.7%) were chosen as combination partner to ADDs to a similar degree as quetiapine (21.2%), whereas the combination with aripiprazole was less common (6.5%). SSNRIs were more commonly combined with SGAs than SSRIs and NaSSAs among all degrees of severity. Furthermore, about 25–30% of all patients treated with ADDs patients received a combination of ADDs. The combination of SSRI or SSNRI with NaSSA was the most used among all degrees of MDD severity. TCAs were used in about 10% of patients treated with SSRI/SSNRIs with moderate and severe MDD. Most commonly, TCAs with sedating properties (e.g., trimipramine, amitriptyline, and doxepin) were combined with SSRIs/SSNRIs (data not shown). Finally, the most frequent triple therapy was the combination of two ADDs and one APD in moderate MDD (9.8%) and ADD + ADP + TRD in severe MDD (14.5%) and psychotic MDD (33.5%; Table 5).

**Table 5:** Percentage of combination and augmentation therapies of antidepressant drugs (ADDs) and of ADD subgroups with other drug groups/single drugs (% of patients exposed to ADD and AD subgroups, respectively) according to severity of major depressive disorder (MDD)*

		Moderate MDD	Severe MDD	Psychotic MDD	Overall: X^2^(2,43,868)	Moderate vs. severe: X^2^(1,38,278)	Moderate vs. psychotic: X^2^(1,17,906)	Severe vs. psychotic: X^2^(1,31,552)
*Combination (% of all patients in each MDD group)*								
ADD	+ ADD	26.3%	29.3%	24.9%	66.0, *p* < 0.001, *V* = 0.03	37.0, *p* < 0.001, ϕ = 0.03	3.9, *p* < 0.05, ϕ = 0.01	43.7, *p* < 0.001, ϕ = 0.04
	+ APD	32.8%	43.6%	74.4%	2714.7, *p* < 0.001, *V* = 018	405.6, *p* < 0.001, ϕ = 0.10	2680.6, *p* < 0.001, ϕ = 0.39	1746.0, *p* < 0.001, ϕ = 0.24
	*Quetiapine*	*12.7%*	*15.9%*	*21.2%*	212.5, *p* < 0.001, *V* = 0.05	67.6, *p* < 0.001, ϕ = 0.04	213.7, *p* < 0.001, ϕ = 0.11	92.3, *p* < 0.001, ϕ = 0.05
	*Olanzapine*	*4.2%*	*6.7%*	*20.5%*	1527.0, *p* < 0.001, *V* = 0.13	94.1, *p* < 0.001, ϕ = 0.05	1212.4, *p* < 0.001, ϕ = 0.26	1054.3, *p* < 0.001, ϕ = 0.18
	*Risperidone*	*2.7%*	*4.5%*	*18.7%*	1998.2, *p* < 0.001, *V* = 0.15	71.8, *p* < 0.001, ϕ = 0.04	1385.8, *p* < 0.001, ϕ = 0.28	1421.7, *p* < 0.001, ϕ = 0.21
	*Aripiprazole*	*1.7%*	*2.7%*	*6.5%*	323.6, *p* < 0.001, *V* = 0.06	36.0, *p* < 0.001, ϕ = 0.03	286.1, *p* < 0.001, ϕ = 0.13	203.8, *p* < 0.001, ϕ = 0.08
	+ FGA	16.7%	22.2%	24.2%	194.7, *p* < 0.001, *V* = 0.05	155.4, *p* < 0.001, ϕ = 0.06	140.3, *p* < 0.001, ϕ = 0.09	10.5, *p* < 0.05, ϕ = 0.02
	+ lp FGA	15.2%	20.2%	17.7%	141.2, *p* < 0.001, *V* = 0.04	138.0, *p* < 0.001, ϕ = 0.06	17.9, *p* < 0.001, ϕ = 0.03	18.1, *p* < 0.001, ϕ = 0.02
	+ TRD	20.0%	26.8%	38.6%	690.1, *p* < 0.001, *V* = 0.09	208.2, *p* < 0.001, ϕ = 0.07	694.7, *p* < 0.001, ϕ = 0.20	311.7, *p* < 0.001, ϕ = 0.10
	+ HYPD	12.7%	14.6%	12.8%	31.1, *p* < 0.001, *V* = 0.02	25.1, *p* < 0.001, ϕ = 0.03	0.0, *p* = 0.85, ϕ < 0.01	12.2, *p* < 0.001, ϕ = 0.02
	+ LI	3.0%	5.7%	6.5%	155.5, *p* < 0.001, *V* = 0.04	132.4, *p* < 0.001, ϕ = 0.06	120.0, *p* < 0.001, ϕ = 0.08	5.4, *p* < 0.001, ϕ = 0.01
	+ AED	11.4%	14.3%	12.0%	69.3, *p* < 0.001, *V* = 0.03	60.7, *p* < 0.001, ϕ = 0.04	1.4, *p* = 0.25, ϕ = 0.01	20.3, *p* < 0.001, ϕ = 0.03
	+ ADD + APD	9.8%	14.1%	21.0%	411.9, *p* < 0.001, *V* = 0.07	139.2, *p* < 0.001, ϕ = 0.06	418.4, *p* < 0.001, ϕ = 0.15	168.8, *p* < 0.001, ϕ = 0.07
	+ APD + APD	4.4%	8.0%	19.5%	1149.7, *p* < 0.001, *V* = 0.11	169.9, *p* < 0.001, ϕ = 0.07	1058.4, *p* < 0.001, ϕ = 0.24	673.7, *p* < 0.001, ϕ = 0.15
	+ APD + TRD	9.2%	14.5%	33.5%	1782.0, *p* < 0.001, *V* = 0.14	210.3, *p* < 0.001, ϕ = 0.07	1625.4, *p* < 0.001, ϕ = 0.30	1131.6, *p* < 0.001, ϕ = 0.19
	+ APD + HYPD	4.9%	7.5%	11.0%	221.3, *p* < 0.001, *V* = 0.05	90.8, *p* < 0.001, ϕ = 0.05	225.6, *p* < 0.001, ϕ = 0.11	75.5, *p* < 0.001, ϕ = 0.05
*Combination (% of all patients treated with SSRI, SSNRI, NaSSA, and TCA)*								
SSRI +	NaSSA	15.2%	20.0%	20.2%	136.5, *p* < 0.001, *V* = 0.04	127.9, *p* < 0.001, ϕ = 0.06	68.9, *p* < 0.001, ϕ = 0.06	0.1, *p* = 0.73, ϕ < 0.01
	SSNRI	1.8%	2.7%	2.7%	30.1, *p* < 0.001, *V* = 0.02	28.8, *p* < 0.001, ϕ = 0.03	15.3, *p* < 0.001, ϕ = 0.03	0.0, *p* = 0.99, ϕ = 0.00
	TCA	9.9%	10.7%	7.7%	46.3, *p* < 0.001, *V* = 0.02	5.7, *p* < 0.05, ϕ = 0.01	22.2, *p* < 0.001, ϕ = 0.04	45.3, *p* < 0.001, ϕ = 0.04
	SGA/TGA	23.9%	30.3%	75.7%	5027.9, *p* < 0.001, *V* = 0.24	168.8, *p* < 0.001, ϕ = 0.07	4296.1, *p* < 0.001, ϕ = 0.49	4010.2, *p* < 0.001, ϕ = 0.36
	FGA	15.9%	21.4%	20.7%	162.8, *p* < 0.001, *V* = 0.04	160.2, *p* < 0.001, ϕ = 0.06	61.6, *p* < 0.001, ϕ = 0.06	1.3, *p* = 0.25, ϕ = 0.01
	lp FGA	14.7%	19.5%	15.8%	147.8, *p* < 0.001, *V* = 0.04	130.6, *p* < 0.001, ϕ = 0.06	3.6, *p* = 0.06, ϕ = 0.01	41.2, *p* < 0.001, ϕ = 0.04
	LI	2.7%	4.3%	5.6%	97.1, *p* < 0.001, *V* = 0.03	58.7, *p* < 0.001, ϕ = 0.04	93.0, *p* < 0.001, ϕ = 0.07	18.0, *p* < 0.001, ϕ = 0.02
SSNRI +	SSRI	0.2%	2.7%	2.6%	280.7, *p* < 0.001, *V* = 0.06	280.7, *p* < 0.001, ϕ = 0.09	235.5, *p* < 0.001, ϕ = 0.11	0.2, *p* = 0.67, ϕ < 0.01
	NaSSA	20.7%	24.7%	24.6%	78.0, *p* < 0.001, *V* = 0.03	74.5, *p* < 0.001, ϕ = 0.04	34.2, *p* < 0.001, ϕ = 0.04	0.0, *p* = 0.99, ϕ = 0.00
	TCA	10.2%	11.0%	6.2%	116.1, *p* < 0.001, *V* = 0.04	5.6, *p* < 0.05, ϕ = 0.01	75.5, *p* < 0.001, ϕ = 0.06	116.2, *p* < 0.001, ϕ = 0.06
	SGA/TGA	31.8%	40.0%	79.2%	3719.4, *p* < 0.001, *V* = 0.21	240.0, *p* < 0.001, ϕ = 0.08	3471.5, *p* < 0.001, ϕ = 0.44	2837.8, *p* < 0.001, ϕ = 0.30
	FGA	17.3%	23.0%	25.1%	204.4, *p* < 0.001, *V* = 0.05	162.7, *p* < 0.001, ϕ = 0.07	147.7, *p* < 0.001, ϕ = 0.09	11.3, *p* < 0.001, ϕ = 0.02
	lp FGA	16.2%	15.9%	20.3%	66.0, *p* < 0.001, *V* = 0.03	0.6, *p* = 0.45, ϕ < 0.01	44.8, *p* < 0.001, ϕ = 0.05	64.1, *p* < 0.001, ϕ = 0.05
	LI	4.7%	7.6%	8.6%	137.5, *p* < 0.001, *V* = 0.04	112.9, *p* < 0.001, ϕ = 0.05	105.0, *p* < 0.001, ϕ = 0.08	6.4, *p* < 0.05, ϕ = 0.01
NaSSA +	SSRI	22.5%	22.3%	20.2%	13.5, *p* < 0.05, *V* = 0.01	0.2, *p* = 0.66, ϕ < 0.01	11.9, *p* < 0.001, ϕ = 0.03	11.8, *p* < 0.001, ϕ = 0.02
	SSNRI	22.6%	26.9%	26.3%	82.6, *p* < 0.001, *V* = 0.03	81.3, *p* < 0.001, ϕ = 0.05	29.1, *p* < 0.001, ϕ = 0.04	0.8, *p* = 0.36, ϕ = 0.01
	TCA	3.6%	5.3%	4.7%	53.4, *p* < 0.001, *V* = 0.02	53.3, *p* < 0.001, ϕ = 0.04	12.3, *p* < 0.001, ϕ = 0.03	3.4, *p* = 0.07, ϕ = 0.01
	SGA/TGA	22.3%	30.2%	75.2%	5166.4, *p* < 0.001, *V* = 0.24	260.6, *p* < 0.001, ϕ = 0.08	4530.6, *p* < 0.001, ϕ = 0.50	3946.5, *p* < 0.001, ϕ = 0.35
	FGA	16.3%	20.5%	21.8%	115.8, *p* < 0.001, *V* = 0.04	95.2, *p* < 0.001, ϕ = 0.05	78.7, *p* < 0.001, ϕ = 0.07	4.7, *p* = 0.03, ϕ = 0.01
	lp FGA	14.6%	18.5%	15.3%	103.1, *p* < 0.001, *V* = 0.03	89.0, *p* < 0.001, ϕ = 0.05	1.5, *p* = 0.22, ϕ = 0.01	32.0, *p* < 0.001, ϕ = 0.03
	LI	3.5%	5.7%	6.6%	107.7, *p* < 0.001, *V* = 0.04	85.2, *p* < 0.001, ϕ = 0.05	86.6, *p* < 0.001, ϕ = 0.07	6.8, *p* < 0.05, ϕ = 0.01
TCA +	SSRI	29.7%	21.7%	15.2%	562.8, *p* < 0.001, *V* = 0.08	290.8, *p* < 0.001, ϕ = 0.09	429.2, *p* < 0.001, ϕ = 0.15	119.0, *p* < 0.001, ϕ = 0.06
	NaSSA	7.3%	9.6%	9.3%	55.6, *p* < 0.001, *V* = 0.03	54.7, *p* < 0.001, ϕ = 0.04	21.1, *p* < 0.001, ϕ = 0.03	0.5, *p* = 0.49, ϕ < 0.01
	SSNRI	22.5%	21.7%	13.2%	230.0, *p* < 0.001, *V* = 0.05	3.1, *p* = 0.08, ϕ = 0.01	211.1, *p* < 0.001, ϕ = 0.11	206.2, *p* < 0.001, ϕ = 0.08
	SGA/TGA	20.6%	28.4%	71.9%	4999.3, *p* < 0.001, *V* = 0.24	264.9, *p* < 0.001, ϕ = 0.08	4359.8, *p* < 0.001, ϕ = 0.49	3772.7, *p* < 0.001, ϕ = 0.35
	FGA	15.9%	20.1%	31.6%	587.1, *p* < 0.001, *V* = 0.08	96.7, *p* < 0.001, ϕ = 0.05	575.3, *p* < 0.001, ϕ = 0.18	352.9, *p* < 0.001, ϕ = 0.11
	lp FGA	13.9%	17.4%	19.3%	105.9, *p* < 0.001, *V* = 0.03	75.1, *p* < 0.001, ϕ = 0.04	85.2, *p* < 0.001, ϕ = 0.07	11.4, *p* < 0.001, ϕ = 0.02
	LI	5.5%	9.1%	11.5%	221.1, *p* < 0.001, *V* = 0.05	148.1, *p* < 0.001, ϕ = 0.06	202.7, *p* < 0.001, ϕ = 0.11	30.7, *p* < 0.001, ϕ = 0.03

*SSRI* selective serotonin reuptake inhibitor, *SSNRI* selective serotonin–norepinephrine reuptake inhibitor, *NaSSA* noradrenergic and specific serotonergic antidepressant, *TCA* tricyclic antidepressant, *APD* antipsychotic drug, *TRD* tranquilizing drug, *HYPD* hypnotic drug, *LI* lithium, *AED* antiepileptic drug, *SGA* second-generation antipsychotic drug, *TGA* third-generation antipsychotic drug, *lp* *FGA* low-potency first-generation antipsychotic drug

*the percentage of patients treated refers to the drug group in the left column of the table

## Discussion

The main focus was to analyze psychopharmacological treatment of psychiatric inpatients in relation to severity of MDD according to the ICD-10 in a naturalistic setting. To the best of our knowledge, this is the first study to provide a detailed analysis of utilization rates of psychotropic drugs in psychiatric inpatients depending on the severity degree of MDD. Most patients in the present study were treated with at least one psychotropic drug, of which ADDs were the most used psychotropic drug group. ADD use differed only slightly between severity levels. The utilization of APDs showed greater differences in association with severity of MDD. While the rates of lp FGA utilization were relatively equal among all three degrees of severity, SGAs were used significantly more often in patients with psychotic MDD. ADD monotherapy was observed in only a small proportion of patients, whereas the use of two or more psychotropic drugs was common even among moderate MDD and was highest among patients with psychotic MDD. The number of psychotropic drugs used per patient also increased with severity of MDD. Patients with psychotic MDD showed the highest utilization of psychotropic drugs and monotherapy was least common among these patients. The combination of two ADDs was observed in almost one-fourth of patients irrespectively of severity degree. The use of an ADD combined with an APD varied significantly according to severity of MDD and was lowest among patients with moderate MDD (32.8%) and highest in psychotic MDD (74.4%).

It is important to note that guidelines are merely able to make recommendations for the treatment that should be offered to the affected individuals. Unless appointed by court order, treatment cannot be forced upon the patient regardless of guideline suggestions. This should be considered whenever the implementation of guideline recommendations is discussed in the following.

### Antidepressant drugs

#### Use of ADDs in the treatment MDD according to severity

The German S3 guideline recommends that patients suffering from moderate MDD should be offered either psychotherapy or an ADD, whereas other international guidelines recommend treatment with an ADD in moderate MDD after first onset of illness (Kennedy et al. 2016; Gelenberg et al. 2010; NICE 2009; Bauer et al. 2013). Accordingly, the high utilization rate of ADDs exceeding 90% of patients with moderate MDD in this study suggests that this group of inpatients experiences a higher subjective burden of illness and/or suffer from a treatment-resistant MDD. During treatment in a psychiatric hospital, an existing psychopharmacological treatment is often enhanced or a new drug is initiated, rather than providing psychotherapy. In German-speaking countries, psychosomatic hospitals are generally better equipped to intensify psychotherapy than psychiatric departments. A recently published study including 14,418 inpatients in Germany describes a similar trend with 87% of individuals with moderate MDD treated with either an ADD or an APD (Wolff et al. 2021).

National and international guidelines recommend that patients suffering from severe or psychotic MDD should be offered a combination of an ADD with psychotherapy (Bauer et al. 2013; Gelenberg et al. 2010; NICE 2009; DGPPN et al. 2015; Kennedy et al. 2016). Therefore, in accordance with these guidelines, all patients with severe or psychotic MDD should be treated with an ADD, which—with an ADD utilization rate of 89.8% (severe MDD) and 87.9% (psychotic MDD)—was not the case in the present study. Depending on the severity level of MDD, between 10.2% and 14.2% of patients were not treated with an ADD. This may be partly due to the manner in which AMSP data are collected. Because AMSP only collects pharmacoepidemiological data on 2 days per year, the data set only allows insight into the prescribing patterns on those individual days and not over the course of treatment over time. As a result, psychopharmacotherapy may not have been initiated at the time of data collection. Alternatively, the individual may have declined the use of an ADD due to its profile of potential ADRs (Bockting et al. 2008) or—especially among patients suffering from psychotic MDD—the use of psychotropic drugs may have been refused due to illness-related, delusional beliefs (e.g., nihilistic delusions, extreme hopelessness, and delusions of poisoning). Switching strategies which involve gradually tapering the first ADD followed by an adequate washout period prior to initiation of treatment with a new ADD to avoid severe ADRs such as serotonin syndrome (Keks et al. 2016) may have further contributed to the under-utilization of ADDs among severely depressed patients.

In general, ADD subgroups with dual action (e.g., NaSSAs or SSNRIs) were used more often in the treatment of severe MDD or psychotic MDD, whereas patients with moderate MDD were more likely to be treated with SSRIs. It has been suggested that two mechanisms of action (e.g., inhibition of both serotonin and norepinephrine transporters by SSNRIs) may be superior in the treatment of MDD (Stahl et al. 2005). Therefore, after first-line treatment with an SSRI as suggested by the WFSBP (Bauer et al. 2013) has failed, physicians and patients may opt for allegedly more efficacious ADDs. Venlafaxine has demonstrated a higher efficacy in the treatment of psychiatric inpatients with severe MDD in comparison to placebo (Guelfi et al. 1995). Furthermore, our results indicate that patients with severe MDD may require ADDs with stronger sedating, sleep-inducing properties such as mirtazapine (Atkin et al. 2018).

Overall, the use of TCAs ranged from 15.7% among patients with severe MDD to 11.9% of patients with moderate MDD. TCAs were only rarely used as monotherapy for the treatment of MDD, suggesting that TCAs were primarily exploited for their sedative properties (Everitt et al. 2014). Emphasizing this theory is the very low usage (i.e., < 2.5%, see Methods) of the non-sedative TCA clomipramine in the present sample. Overall, the use of TCAs in the treatment of MDD significantly decreased during the respective time period of this study (Seifert et al. 2021a).

Although the prevalence of treatment-resistant depression has been estimated to be 13–31% within the inpatient setting (Gronemann et al. 2018) and that MAO-Is are considered particularly effective in the treatment of patients suffering from MDD subtypes that are difficult to treat such as treatment-resistant or atypical depression (Shulman et al. 2013), MAO-Is were rarely used in the present study. Despite their efficacy, guidelines list the utilization of MAO-Is as a second- or third-line option (Shulman et al. 2013; Kennedy et al. 2016; Gelenberg et al. 2010; NICE 2009). Their successful implementation requires a high level of patient adherence due to the necessary tyramine restrictive diet and possible ADRs, especially under treatment with the irreversible MAO-I tranylcypromine (Shulman et al. 2013; Kennedy et al. 2016; Gelenberg et al. 2010; NICE 2009).

#### Dosing of ADDS in the treatment MDD according to severity

Dosing of SSRIs, SSNRIs, and TCAs correlated with severity of MDD. Although previous studies have concluded that SSRIs tend to show a flat dose–response curve, a systematic review from 2016 showed that the use of higher dosages of SSRIs as a group has been associated with a slightly higher efficacy in the treatment of MDD (Jakubovski et al. 2016). Higher doses in concordance with severity were detected for patients treated with both citalopram and escitalopram but not sertraline in this analysis.

Current literature suggests a more clear-cut positive dose–response for SSNRIs (Bech et al. 2006). This especially applies to venlafaxine for which the reuptake of noradrenaline is observed when applied at dosages above 75 mg per day (Blier et al. 2007). This does not adequately explain the dose difference among patients treated with venlafaxine in this study as the dose requirement for noradrenergic reuptake inhibition was met in patients of all severity levels. However, the use of venlafaxine doses between 225 and 300 mg per day has been associated with an additional mechanism of action, namely the reuptake of dopamine (Raouf et al. 2017). A more balanced reuptake inhibition of serotonin and noradrenaline has been described for duloxetine. The optimal effective dose of duloxetine is 60 mg, but higher doses of 80 or 120 mg may be observed after a longer treatment period (Bech et al. 2006).

TCAs are increasingly used for indications other than MDD (Noordam et al. 2015) such as headache prophylaxis (Jackson et al. 2010), neuropathic pain disorders (Sindrup et al. 2005), and sleeping disorders (Cassano and Fava 2004), which typically require the use of doses lower than for the treatment MDD (Jackson et al. 2010; Sindrup et al. 2005). Though the required doses are poorly defined, most commonly doses greater than 100 or 125 mg per day are recommended for the treatment of MDD (Furukawa et al. 2002). This could in part explain the application of significantly higher doses of TCAs (i.e., amitriptyline, doxepin, and trimipramine) among patients with severe or psychotic MDD in this study, whereas patients with moderate MDD received an average of 75 mg of each of these TCAs. On the other hand, the use of low-dose TCAs in the treatment of MDD is justified, although it may not be as effective as standard dosage or TCAs (Furukawa et al. 2002). In general, the use of higher doses of any ADD may be offset by a lower tolerability due to ADRs (Jakubovski et al. 2016; Furukawa et al. 2002).

### Use of non-ADDs in the treatment of MDD

Official guideline recommendations for the treatment of MDD favor monotherapy with an ADD unless certain prerequisites are met (Kennedy et al. 2016; Bauer et al. 2013; NICE 2009; DGPPN et al. 2015; Gelenberg et al. 2010). In this study, ADD monotherapy was applied in approximately one-fourth of patients with moderate MDD, less than one-fifth of patients with severe MDD and only 4.4% of patients with psychotic MDD. Thus, most patients were treated with adjunctive psychotropic drugs. The use of several psychotropic drug groups such as AEDs, lp FGAs, and HYPDs was distributed relatively equally among all severity degrees, indicating that these drug groups may primarily be used to treat underlying comorbidities (e.g., epilepsy) or common symptoms of MDD that occur among all degrees of severity (e.g., insomnia, anxiety) and not necessarily as augmentation strategies.

#### Antipsychotic drugs

APD augmentation is known to be effective in treating patients with treatment-resistant MDD (Zhou et al. 2015; Cantù et al. 2021) and is recommended by several guidelines for the treatment of psychotic MDD from the beginning of therapy (Bauer et al. 2013; Gelenberg et al. 2010; Kennedy et al. 2016). This strategy can be potentially problematic due to the higher risk of ADRs (Zhou et al. 2015). Overall, APDs are not recommended for routine clinical use (Mulder et al. 2018), but the combination of APDs with ADDs has been found to be superior in the treatment of psychotic MDD than either drug group alone (Farahani and Correll 2012; Wijkstra et al. 2015). The duration of APD treatment should be as short as possible but as long as necessary (Mulder et al. 2018), since definitive recommendations are still lacking (Strawbridge et al. 2019). In the present study, the combination of an ADD with an APD was the most commonly used combination of psychotropic drugs in patients suffering from MDD regardless of severity. While information on treatment resistance is not available, it can be assumed that even patients with moderate MDD in the inpatient setting experience treatment resistance, therefore warranting this treatment strategy. Individual comorbidities or a specific clinical presentation of symptoms of patients with MDD may further necessitate the use of APDs irrespectively of severity. For example, APDs may alleviate symptoms such as severe rumination or melancholia (Mulder et al. 2018). However, in the present study, patients remain more likely to be treated with an APD if they are more severely affected. A majority of patients suffering from psychotic MDD were additionally treated with an APD, especially an SGA. The use of APDs in the treatment of patients with has become more common over time (Seifert et al. 2021a). Konstantinidis et al. were able to detect a significant increase in the usage of SGAs in inpatients with mild-to-moderate depression which was previously documented from 2000 (32.0%) and 2007 (41.0%). Meanwhile, APD use in the treatment of severely depressed patients without psychotic symptoms increased from 14.86% (2000) to 29.45% (2007) (Konstantinidis et al. 2012).

Moreover, APDs, in particular SGAs, have potential antidepressant effects mediated by a variety of neurotransmitters including serotonin, glutamate, gamma-aminobutyric acid (GABA), and cortisol and neurotrophic factors (Wang and Si 2013). While APDs are not approved as monotherapy in MDD (DGPPN et al. 2015) and proof of efficacy as monotherapy is lacking (Komossa et al. 2010), APD monotherapy was observed in about 2% of patients with moderate and severe MDD and 3% of patients with psychotic MDD. This observation may also be the result of switching strategies.

Quetiapine extended-release, when used concomitantly with an ADD, is the only APD approved for treatment of MDD in Germany. In the present study, quetiapine was the most frequently used APD alongside an ADD. Besides quetiapine, the combination of the two SGAs olanzapine and risperidone with an ADD showed high utilization rates among patients suffering from psychotic MDD, presumably due to the strong antipsychotic properties of these two APDs. Some APDs are considered especially effective in the augmentation therapy of specific symptoms in patients suffering from MDD. For example, the augmentation of fluoxetine with olanzapine, which has been approved by the U.S. Food and Drug Administration (FDA) as adjunctive therapy for treatment-resistant depression, showed higher response rates than either drug alone (Ng et al. 2006). The rapid onset of improvement was ascribed to the possible attenuation of symptoms like agitation and anxiety (Ng et al. 2006). Zhou et al. found that the adjunctive use of risperidone showed statistically significant benefits in functioning and quality of life in the treatment of MDD in comparison to placebo (Zhou et al. 2015). Overall, evidence for the efficacy of SGAs such as risperidone and olanzapine in the treatment of MDD is not as robust as for quetiapine, so that further research is needed (Cantù et al. 2021).

While the utilization of hp FGAs—rarely used in general—also differed significantly according to the severity of MDD, lp FGA use showed much less deviation in relation to severity. Overall, the use of lp FGAs appears to be indispensable in the treatment of MDD and has shown only minimal changes over the years (Konstantinidis et al. 2012). Lp FGAs are often prescribed for their sedating properties as an alternative to benzodiazepines, Z-drugs, or other sedative drugs with a risk for addiction. While all APDs are generally associated with an increased likelihood of sedation, lp FGAs are not associated with the same debilitating ADRs such as metabolic syndrome and are less likely to induce extrapyramidal motors symptoms (Muench and Hamer 2010) making their use appealing regardless of severity.

#### Lithium

Though recommended as a first-line treatment strategy for patients with treatment refractory MDD (Bauer et al. 2013; Gelenberg et al. 2010; NICE 2009; DGPPN et al. 2015; Kennedy et al. 2016), the combination of an ADD with LI was used in only 5.7% of patients suffering from severe MDD and therefore significantly less often than expected in this study. As previously described, the use of lithium in the treatment of inpatients with MDD significantly decreased from 2001 to 2017 (Seifert et al. 2021a). Lithium is the only drug with verified anti-suicidal efficacy approved for use in affective disorders (Del Matto et al. 2020; Bschor 2014; Barroilhet and Ghaemi 2020). Lithium is significantly more effective in the prevention of relapses requiring hospitalization than treatment with ADDs (Bschor 2014). However, lithium's well-established benefits may be overshadowed by several practical issues. Before initiation of treatment and in regular intervals during treatment, several medical examinations including kidney function (creatinine clearance and glomerular filtration rate) and thyroid function are required. However, it should also be acknowledged that the use of other drugs with mood-stabilizing properties such as valproate and carbamazepine entail similarly complex monitoring (Ng et al. 2009). Due to the narrow therapeutic window of lithium and the higher risk of intoxication, patient compliance and careful monitoring of serum lithium levels is a prerequisite of successful lithium therapy (Bschor 2014). Furthermore, the long-term use of lithium is associated with major health complications such as advanced kidney disease and thyroid dysfunction (Shine et al. 2015). These considerations may significantly contribute to a patient’s and physician’s reluctance to initiate treatment with lithium as has also been observed by others (Bschor 2014).

#### Tranquilizing drugs

Benzodiazepines do not exert antidepressant effects and are not approved for the treatment of MDD (DGPPN et al. 2015). Nonetheless, they are often used with the intention of bridging the effect of ADDs to treat acutely occurring symptoms such as suicidality, anxiety, restlessness, or sleep disturbances, and may also improve response and lower ADD discontinuation rates at least in the short term (DGPPN et al. 2015). Utilization of TRDs was related to severity of MDD—more than 40% of patients with psychotic MDD were treated with TRDs, whereas this applied to only about one-fifth of patients with moderate MDD. An association between the use of benzodiazepines and severity of symptoms has been determined by some (Furukawa et al. 2002; Dold et al. 2020) and questioned by others (Rizvi et al. 2015).

### Polypsychopharmacotherapy

A majority of patients was treated with more than one psychotropic drug. While an ADD and an APD were the most frequently concomitantly used psychotropic drugs (see below), the use of two ADDs was relatively equal regardless of severity of MDD. According to the German S3 guideline from 2015, one of the treatment options in patients who have not sufficiently responded to ADD monotherapy is a combination of two ADDs, for example, an SSRI/SSNRI and mirtazapine (DGPPN et al. 2015). Although this combination is not supported by high-quality evidence (Connolly and Thase 2011; Kessler et al. 2018), the present study found that this combination was the most frequently used combination of ADDs in all three severity groups. This may be due to the complementary effects of mirtazapine to SSRIs or SSNRIs (e.g., attenuation of sexual dysfunction (Rizvi and Kennedy 2013)). The combination of an SSNRI with mirtazapine was also encountered relatively frequently in an inpatient setting in the study conducted by Wolff et al. (Wolff et al. 2021). The simultaneous use of both an SSRI and an SSNRI did not exceed 3% among any severity degree and can probably be attributed to cross-tapering of ADDs.

Rarely used in ADD monotherapy, TCAs were most commonly combined with SSRIs and SSNRIs. In almost 30% of patients with moderate MDD treated with a TCA, it was combined with an SSRI. The use of a TCA with an SSRI or an SSNRI decreased with increasing degree of severity, although it has been suggested that this somewhat controversial combination strategy may more rapidly reduce depressive symptoms in psychiatric inpatients (Sonawalla and Fava 2001). This frequently used drug combination is associated with several potentially life-threatening pharmacodynamic drug–drug interactions such as serotonin syndrome—especially when combining TCAs with serotonin reuptake inhibitory properties (e.g., clomipramine) with SSRI/SSNRIs (Gillman 2007; Chan et al. 1998)—and an increased risk of QTc prolongation (Wenzel-Seifert et al. 2011; Schächtele et al. 2014). Additionally, pharmacokinetic drug–drug interactions should be considered. Depending on the substance used, several SSRI/SSNRIs inhibit metabolizing cytochrome P450 isoenzymes and therefore result in substantially increased TCA plasma concentrations with a greater risk of ADRs (Taylor 1995).

The opposite utilization trend was observed for the simultaneous use of a TCA with an SGA which increased in concordance to the severity degree. Overall, the proportion of patients receiving more than two psychotropic drugs greatly exceeded the percentage of patients with monotherapy. Several other studies examining psychopharmacotherapy of psychiatric inpatients within the AMSP database also found high rates of polypharmacotherapy among patients with post-traumatic stress disorder (Reinhard et al. 2020), schizophrenia (Toto et al. 2019), and bipolar disorder (Haeberle et al. 2012), suggesting that combinations of psychotropic drugs are the rule rather than the exception among psychiatric inpatients. Hahn and colleagues reported similar observations without consideration of the psychiatric diagnosis in 2008 (Hahn et al. 2013): 47.8% of patients were treated with over three psychotropic drugs at admission. Interestingly, a recent Chinese study published in 2019 found that the number of psychotropic drugs used at baseline showed an inverse correlation with treatment response and remission within a naturalistic inpatient sample, implying that while polypharmacy is a proxy for treatment resistance, it does not necessarily generate better treatment outcomes (Chae et al. 2019).

The use of combination therapies is not limited to the inpatient sector as many authors have also found a high proportion of multiple psychotropic drug use among ambulatory patients [e.g., (Glezer et al. 2009; Rhee and Rosenheck 2019; Huang et al. 2018)]. The high proportion of combination therapies possibly suggests that drugs with a single mechanism of action may generate only mediocre outcomes (Millan 2014). While multi-target ADDs that combine two or more complementary mechanisms such as TCAs are used less frequently, their mode of action may be mimicked by combining multiple drugs that act on a variety of neurotransmitters (Millan 2014). The advantage of this strategy is that the receptor profile can be “tailored” to suit specific patient needs, while unwanted effects (e.g., anticholinergic properties) can ideally be avoided. However, the extent of this presumed specificity is severely limited, because many drugs, especially APDs, act on a variety of different receptor systems (e.g., dopamine, histamine, serotonin, and acetylcholine) (Siafis et al. 2018). Further overshadowing the potential benefits of this technique is the simple fact that polypharmacy is not considered more efficacious than monotherapy (Stassen et al. 2021) and is also is a well-established risk factor for ADRs (Zopf et al. 2008).

### Drawbacks and limitations of treatment guidelines

The data presented here highlight significant discrepancies between guideline recommendations and real-world drug utilization. Current guideline recommendations are based on highly selective evidence, mostly deriving from randomized controlled trials (RCTs), systematic reviews, meta-analyses, and Cochrane analyses. RCTs generally include a collective of “ideal” patients that meet a number of inclusion criteria and therefore insufficiently include older and/or multimorbid adults (Clark et al. 2019; Hanlon et al. 2019) let alone represent the socioeconomic, ethnic, and immigrant backgrounds (Polo et al. 2019), many of which are frequently encountered in a “real life” psychiatric inpatient setting (Patten 2017; Read et al. 2017; Bas-Sarmiento et al. 2017). As a result, evidence from RCTs does not necessarily reflect the actual needs of psychiatric patients. “Real world data” as provided by pharmaoepidemiological data such as the data acquired by AMSP is essential in understanding the needs of “real” patients versus the pre-selected test subjects included in RCTs. For this reason, evidence originating from controlled RCTs as well as uncontrolled “real world evidence” should find consideration in future treatment guidelines.

## Strengths and limitations

The data presented here provide a descriptive analysis of the current state of drug utilization based on severity of MDD during a 17-year time period and includes a very large study population. Overall time trends and sex differences in the utilization of psychotropic drugs in patients with MDD have been described in detail elsewhere (Seifert et al. 2021a, 2021b). Due to the inpatient setting, AMSP is able to assess actual utilization rates of psychotropic rates versus merely prescription rates as is the case in most ambulatory study populations. At the same time, AMSP collects naturalistic, real-world drug use data, therefore acting as a counterpart to data gathered in experimental settings.

Several limitations must also be discussed. Due to the nature of AMSP’s data collection, the reason for the selection of a certain drug/drug group cannot be assessed for this collective of psychiatric inpatients. Therefore, clinically relevant aspects that may also influence the selection of a certain psychotropic drug (e.g., comorbidities indicating the use of a psychotropic drug or other sociodemographic characteristics) were not considered. Also, information in relation to the course of illness and clinical symptoms, the occurrence of ADRs, as well as the use of non-pharmacological treatment options (e.g., psychotherapy and ECT) was not collected. The use of cross-tapering strategies may have led to an overestimation of polypsychopharmacy. A previously established psychopharmacotherapy of patients admitted to inpatient care is often subject to change both in the type and number of psychotropic drugs used. The existing treatment with an ADD may be the first to be adjusted using cross-tapering strategies regardless of severity of MDD, therefore partly accounting for the equal utilization of two ADDs among all degrees of severity.

## Conclusion

Overall, a majority of psychiatric inpatients with MDD were treated with at least one psychotropic drug, most commonly ADDs. The use of multiple psychotropic drugs, especially a combination with APDs, was highly prevalent among all patients irrespective of severity of MDD. This may indicate the need for a rapid improvement of symptoms of MDD within the inpatient setting, that inpatients might be treatment-resistant, or that many patients are inadequately treated with ADD monotherapy. Though approved for treatment-resistant MDD, lithium was underused. While it is not reflected by current guideline recommendations and officially approved indications, it appears APDs and polypsychopharmacotherapy have a substantial standing in the treatment of non-severe MDD and severe MDD with and without psychotic symptoms in psychiatric inpatients.
